# Microbubbles in macrocysts – Contrast-enhanced ultrasound assisted sclerosant therapy of a congenital macrocystic lymphangioma: a case report

**DOI:** 10.1186/s12880-017-0213-9

**Published:** 2017-07-06

**Authors:** Carlos Menendez-Castro, Maren Zapke, Fabian Fahlbusch, Heiko von Goessel, Wolfgang Rascher, Jörg Jüngert

**Affiliations:** Department of Pediatrics and Adolescent Medicine, University Hospital of Erlangen, Loschgestrasse 15, D-91054 Erlangen, Germany

**Keywords:** Contrast agent-enhanced ultrasound, CEUS, Congenital cystic lymphangioma, SonoVue®, Sclerosant therapy, OK-432, Picibanil

## Abstract

**Background:**

Congenital cystic lymphangiomas are benign malformations due to a developmental disorder of lymphatic vessels. Besides surgical excision, sclerosant therapy of these lesions by intracavitary injection of OK-432 (Picibanil®), a lyophilized mixture of group A Streptococcus pyogenes, is a common therapeutical option. For an appropriate application of OK-432, a detailed knowledge about the structure and composition of the congenital cystic lymphangioma is essential. SonoVue® is a commercially available contrast agent commonly used in sonography by intravenous and intracavitary application.

**Case presentation:**

Here we report the case of 2 month old male patient with a large thoracic congenital cystic lymphangioma. Preinterventional imaging of the malformation was performed by contrast-enhanced ultrasound after intracavitary application of SonoVue® immediately followed by a successful sclerotherapy with OK-432.

**Conclusions:**

Contrast agent-enhanced ultrasound imaging offers a valuable option to preinterventionally clarify the anatomic specifications of a congenital cystic lymphangioma in more detail than by single conventional sonography. By the exact knowledge about the composition and especially about the intercystic communications of the lymphangioma sclerosant therapy becomes safer and more efficient.

## Background

Congenital cystic lymphangioma (CCL) is defined as a congenital tumorous formation of lymphatic vessels. About 60% of all lymphangiomas occur at birth, 80–90% before the age of two years. The most frequent localization of CCL is the neck and head region [1]. The fact that the lesions usually have no spontaneous regression, tend to augment in size and can cause life-threatening complications such as occlusion or infiltration of neighbouring organs and structures, underlines the need of an early adequate therapy of lymphangiomas. Surgical excision used to be the first-line treatment of macrocystic lymphangiomas. However, complete excision often is not possible. If the tumor is excised only partially, the recurrence rate is significantly increased [2].

Sclerosant therapy is an alternative to surgical excision, especially in the case of macrocystic lymphangiomas with communicating cysts. Among the different sclerosant agents, OK-432 (Picibanil®) has become the favorite preparation since no perilesional fibrosis occurs after treatment [3]. It is a lyophilized mixture of group A Streptococcus pyogenes cells, which were preincubated with Penicillin G. The efficacy of lymphangioma sclerosant therapy with OK-432 in children has been proven by several clinical studies [3].

Before the injection of OK-432, conventional sonography is required to depict localization, size and structure of the tumor. But one crucial characteristic of CCL, the communication between the cysts, cannot be evaluated sufficiently by regular B-scan ultrasound or by native CT and MRI. Radioscopy with injection of contrast agent, a diagnostic option to test intercystic communication, does not seem appropriate in children because of the radiation exposure.

To our knowledge we report here for the first time a case of preinterventional contrast-enhanced ultrasound (CEUS) of a macrocystic congenital lymphangioma as an alternative method to examine the communication between lesional cysts. Standardized preinterventional usage of CEUS would help to improve the sclerosant therapy of CCL by avoiding unnecessary multiple punctions, and would offer the possibility to reduce the amount of administered OK-432.

## Case presentation

**Fig. 1:**
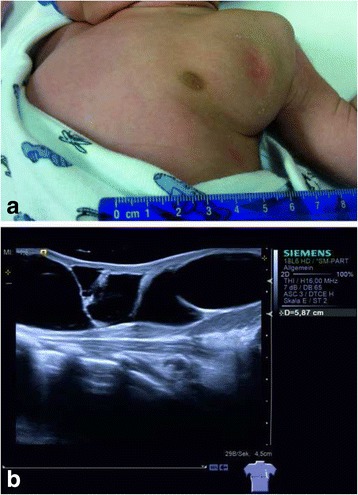
Large congenital macrocystic lymphangioma in the area of the left axilla. **a** Clinical, macroscopic aspect. **b** B-mode ultrasound showing the typical finding of a subcutaneous tumor with multiple homogenous anechoic cysts

**Fig. 2:**
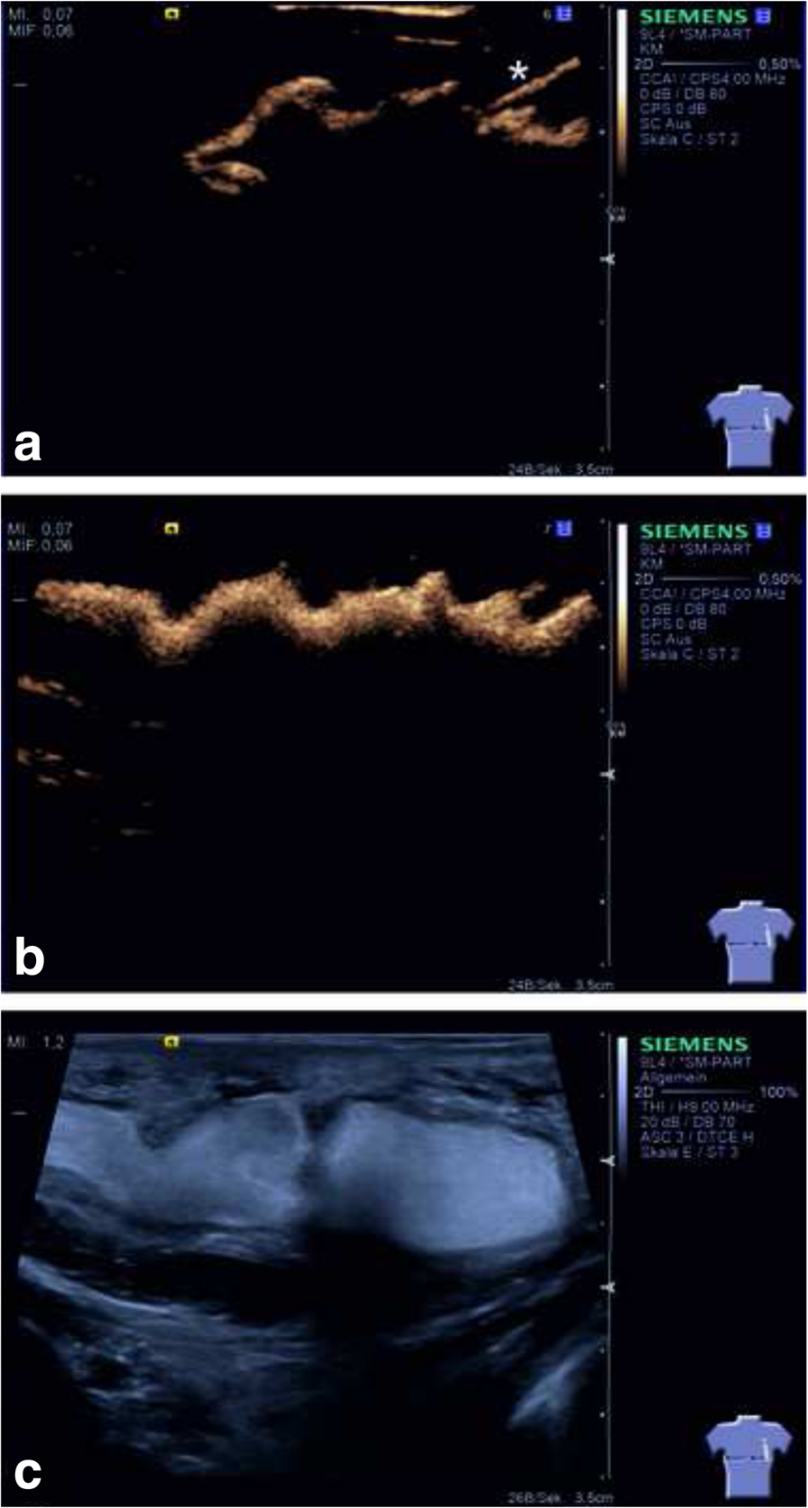
B-Mode contrast agent-enhanced ultrasound of the congenital macrocystic lymphangioma. **a** Early phase of instillation of SonoVue® via an intralesional catheter (*). Microbubbles in the upper part of the cysts. **b** Early depiction of the intercystic communication by homogenous diffusion of microbubbles in the distinct cysts. **c** High resolution B-mode ultrasound showing cysts totally filled with microbubbles

**Fig. 3:**
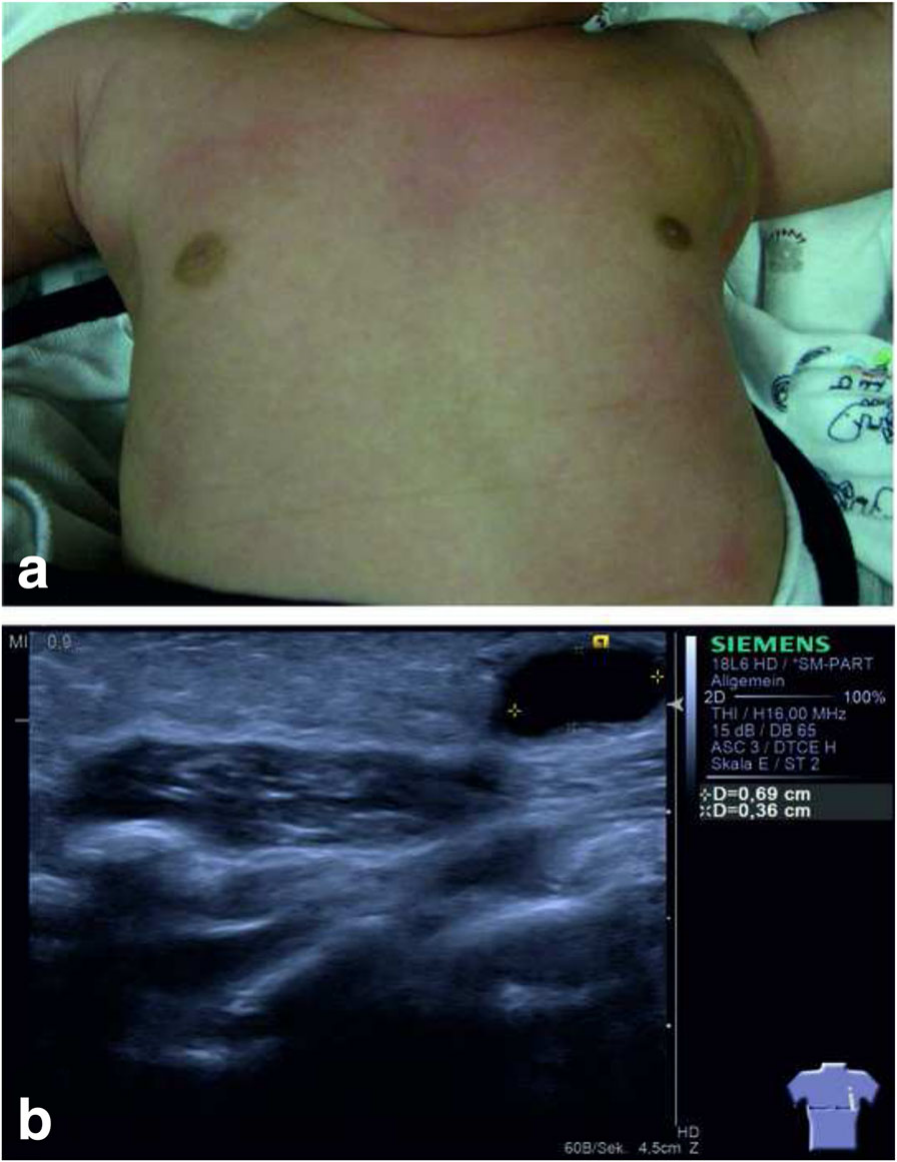
**a** Nearly total remission of the tumor three weeks after sclerosant therapy. **b** B-Mode ultrasound showing a singular small anechoic cyst as a residuum of the CCL 8 weeks after sclerosant therapy

A male Caucasian term newborn (Table 1) presented with a soft tumor of the left axilla with a size of 6.5 × 6.0 cm (Fig. 1a). Sonographically the tumor showed the typical signs of a macrocystic lymphangioma with multiple cysts of a diameter up to 3 cm (Fig. 1b). Color doppler imaging did not reveal perfusion as a sign of combined hemangioma. A spontaneous augmentation in size occurred at the age of 4 weeks. MRI confirmed the diagnosis of a CCL without intrathoracic expansion. Since the axillary swelling persisted until the age of 2 months, sclerotherapy was indicated. Treatment was performed under mild anesthesia with ketamine and propofol in an aseptic environment. SonoVue®-supported CEUS was performed using the linear probe 9 L4 on a Siemens S2000 system equipped with the “Cadence Pulse Sequencing” (CPS) technology at low mechanical index (MI). After aspiration of the cystic fluid, 0.1 ml of SonoVue®, a dispersion of phospholipid-stabilized microbubbles containing sulfurhexafluorid, and 4 ml of sodium chloride 0.9% were injected into the lymphangioma via an intralesional 18 Charrière catheter. (Fig. 2a). The connection between the cysts was proved by depiction of a homogenous diffusion of SonoVue® in the whole tumor (Fig. 2b, c). Before sclerotherapy the injected contrast agent and liquid content of the cysts were aspirated. Then a single injection of OK-432 (0.01 mg/ml sodium chloride 0.9%) was performed via the same catheter. Expectedly one day after intervention fever and a local swelling occurred. C-reactive protein (CRP) increased up to 104 g/dl, normalizing five days after injection. An antibiotic therapy with piperacilline and tobramycin was applied for seven days. Sonographic follow-up examinations showed that the cysts became more solid, accompanied by a subcutaneous edema. Three weeks after intervention we saw a significant involution of the lymphangioma (Fig. 3a). After eight weeks only few singular cysts up to 3 mm (Fig. 3b) could be depicted by sonography.

**Table 1:** Timeline

08/2013	Male term newborn with a congenital soft tumor of the left axilla, postnatal sonography: see Fig. 1a, b
09/2013	Spontaneous augmentation of the tumor, MRI: diagnosis of a CCL without intrathoracic expansion
10/2013	SonoVue® supported CEUS and sclerotherapy of CCL with OK-432
11/2013	Follow up examination by sonography: see Fig. 3a
12/2013	Follow up examination by sonography: see Fig. 3b

## Discussion

We report the successful intracavitary use of CEUS to elucidate intercystic communications in a macrocystic congenital lymphangioma prior to sclerosant therapy with OK-432.

Among the cavernous and capillary lymphangiomas, the cystic lymphangioma is the leading lymphangioma subtype. It is characterized by dilated lymphatic ducts coated by an endothelial layer [1]. Besides surgical resection, sclerosant therapy with OK-432 is a well described and validated therapeutic option for macrocystic congenital lymphangiomas. The lyophilized mixture of group A streptococcus pyogenes induces a local inflammatory reaction, thus reducing the production of the lymphatic fluid and improving its drainage. Complete aspiration of cystic contents seems to be crucial for the success of OK-432 sclerotherapy of macrocystic lymphangiomas [4].

Conventional sonography is a radiation-free diagnostic tool to describe location, size and structure of the lymphangioma. It combines the main advantages of sonographic imaging like mobility, realtime assessment and high spatial resolution. Yet, for sclerosant therapy it is necessary to gain knowledge about the intercystic communication of the tumor. In this context CEUS provides a valuable diagnostic tool.

By preinterventional CEUS we were able to prove extensive intercystic communication in the lymphangioma and thereby to avoid multiple punctions. Thus, not only the risk of periinterventional infection can be reduced, but also the amount of administered OK-432 can be individually case-adapted. Furthermore by utilizing CEUS we were able to verify the proper intracystic positioning of the catheter prior to the injection of the sclerosant agent, which significantly reduces the risk of para-tumorous application of OK-432.

While the utilization of CEUS with SonoVue® was approved for diagnostic liver imaging in children in 2016 in the USA, the utilization of CEUS with SonoVue® in children is still off-label in Europe. There is a long history of safe use of SonoVue® in echocardiography [5] and recent clinical studies document the drug safety of SonoVue® in children [6, 7]. Thus, in our opinion, the advantages of preinterventional CEUS in CCL, including improved application guidance of the sclerosant agent and reduced risk of periinterventional infection due to unnecessary punctions, justify off-label use of SonoVue® in CCL in children. The use of SonoVue® is still quite expensive but this might be compensated by the advantage of a case-adapted and therefore dosage-reduced application of OK-432.

## Conclusions

In summary, this case report shows that contrast-enhanced ultrasound with intracavitary application of SonoVue® might be a helpfull diagnostic technique to depict the communications of cysts in CCL and to optimize sclerotherapy with OK-432 in children. Further clinical studies are needed to analyze the benefits and limits of this procedure.

## Abbreviations

CCL: Congenital cystic lymphangioma
CEUS: Contrast-enhanced ultrasound
CPS: Cadence pulse sequencing
CRP: C-reactive protein
